# The Role of Proximal Femoral Osteotomy for the Treatment of Avascular Necrosis: A Systematic Review of Clinical and Patient-Reported Outcomes

**DOI:** 10.3390/jcm14155592

**Published:** 2025-08-07

**Authors:** Paul L. Rodham, Jamila Tukur Jido, Hannah Bethell, Vasileios P. Giannoudis, Michalis Panteli, Nikolaos K. Kanakaris, Peter V. Giannoudis

**Affiliations:** 1 Leeds Major Trauma Centre, Leeds Teaching Hospital NHS Trust, Leeds LS7 4SA, UK; 2 Academic Department of Trauma and Orthopaedics, Leeds Institute of Rheumatic and Musculoskeletal Medicine, University of Leeds, Leeds LS2 9LU, UK; 3 NIHR Leeds Biomedical Research Centre, Chapel Allerton Hospital, Leeds LS7 4SA, UK; 4 Foundation School, Queen Elizabeth Hospital, Glasgow G4 0SF, UK; 5 School of Medicine, University of Leeds, Leeds LS2 9JT, UK; 6 Royal Devon and Exeter NHS Foundation Trust, Exeter EX2 5DW, UK

**Keywords:** avascular necrosis, osteonecrosis, femoral head, osteotomy, total hip replacement

## Abstract

**Background/Objectives**: Avascular necrosis of the femoral head is a debilitating condition that, if left untreated, leads to progressive arthritis necessitating total hip replacement (THR). In the younger adult population, there is a drive towards joint-preserving procedures, particularly where alternative techniques such as core decompression or vascularised bone grafting are anticipated to fail. Proximal femoral osteotomy is a technique that aims to remove the necrotic segment from the weight bearing area. The presented review aims to examine the efficacy of this technique in the management of avascular necrosis of the femoral head, reporting both rates of conversion to total hip replacement and patient reported outcomes. **Methods**: This systematic review was conducted according to PRISMA guidelines. A search was conducted of PubMed, Ovid Medline, EMBASE, and the Cochrane Library using pre-defined search terms. Data were extracted, and descriptive data presented. Quality of each study was assessed using the NIH quality assessment tool for case series studies. **Results**: Fifty-three studies with data for 2686 osteotomies are presented. Progression of radiological arthrosis was present in 40% of cases, with 20.3% of patients having undergone conversion to THR at a mean of 75.4 months (range 20–132 months). Patient-reported outcome measures were recorded in 1416 patients, of which the Harris Hip Score was the most commonly utilised. This score improved from a mean of 58.3 to 84.4 at a mean follow-up of 102 months. **Conclusions**: Osteotomy represented a valid head-preserving technique in the armamentarium against avascular necrosis of the femoral head, with conversion to THR required in 20.3% of patients at 7 years. In those patients who did not require THR, PROMS were similar to the arthroplasty population.

## 1. Introduction

Avascular necrosis of the femoral head (AVNFH) occurs secondary to aseptic necrosis of osteocytes, culminating in subchondral bone collapse and loss of the structural support for cartilage [1]. This occurs following a disruption to the blood supply to the femoral head, be that traumatic or non-traumatic in origin [2]. In adult populations, the most common causes of osteonecrosis are prolonged treatment with corticosteroids, alcohol misuse, and post-trauma [3,4,5]. Clinically, the presentation of AVNFH varies, though groin pain and reduced range of hip movement are common features [6]. Diagnosis is made in most instances with Magnetic Resonance Imaging (MRI) which possesses a sensitivity of 90–100% [7].

The management of AVNFH remains a topical and evolving area. Some propose early non-operative measures to reduce the need for surgical intervention, whilst others recommend early surgical intervention with the aim of joint preservation [2]. Where joint-preserving procedures are selected, common techniques include core decompression, vascularised bone grafting, and osteotomy [8]. Arthroplasty is often reserved for those patients in whom there is femoral head collapse and secondary arthritic changes [9].

Various osteotomy techniques are employed in the management of AVNFH, including rotational, varus, and valgus osteotomies; all aim to remove the necrotic area from the weight bearing area, and both reduce stresses and improve local perfusion to the necrotic area [10]. The current literature emphasises the importance of patient selection with careful consideration of age, stage, and degree of involvement. In the majority of cases, studies examining outcomes following osteotomy for AVNFH utilise outcome measures including pain relief, radiological union, and delay of total joint replacement [11]. More recently there has been a progression towards increased utilisation of patient-reported outcome measures (PROMS) composed of self-reported questionnaires assessing the patient’s perception of treatment. These measures can more accurately assess those functional outcomes most important to patients, providing a more valuable insight into the results of the treatment strategies proposed [12].

This systematic review aims to examine the role of proximal femoral osteotomy in the management of AVNFH, describing the rate of progression to total hip replacement (THR) and the current reported PROMS.

## 2. Materials and Methods

In order to review outcomes of osteotomy in AVN of the femoral head, a systematic review was conducted in accordance with the guidance described in the Cochrane handbook for systematic reviews, presented in accordance with PRISMA guidelines, and registered with the international prospective register of systematic reviews (PROSPERO-ID 1056765) [13,14].

### 2.1. Outcome Measure

The primary outcome of the presented review was to report rates of conversion to THR in patients undergoing osteotomy as a treatment of hip AVN. The review sought to summarise types of osteotomies performed, post-operative rehabilitation regimes utilised, and secondary outcomes including peri-operative complications and PROMs.

### 2.2. Literature Search

**Figure 1:**
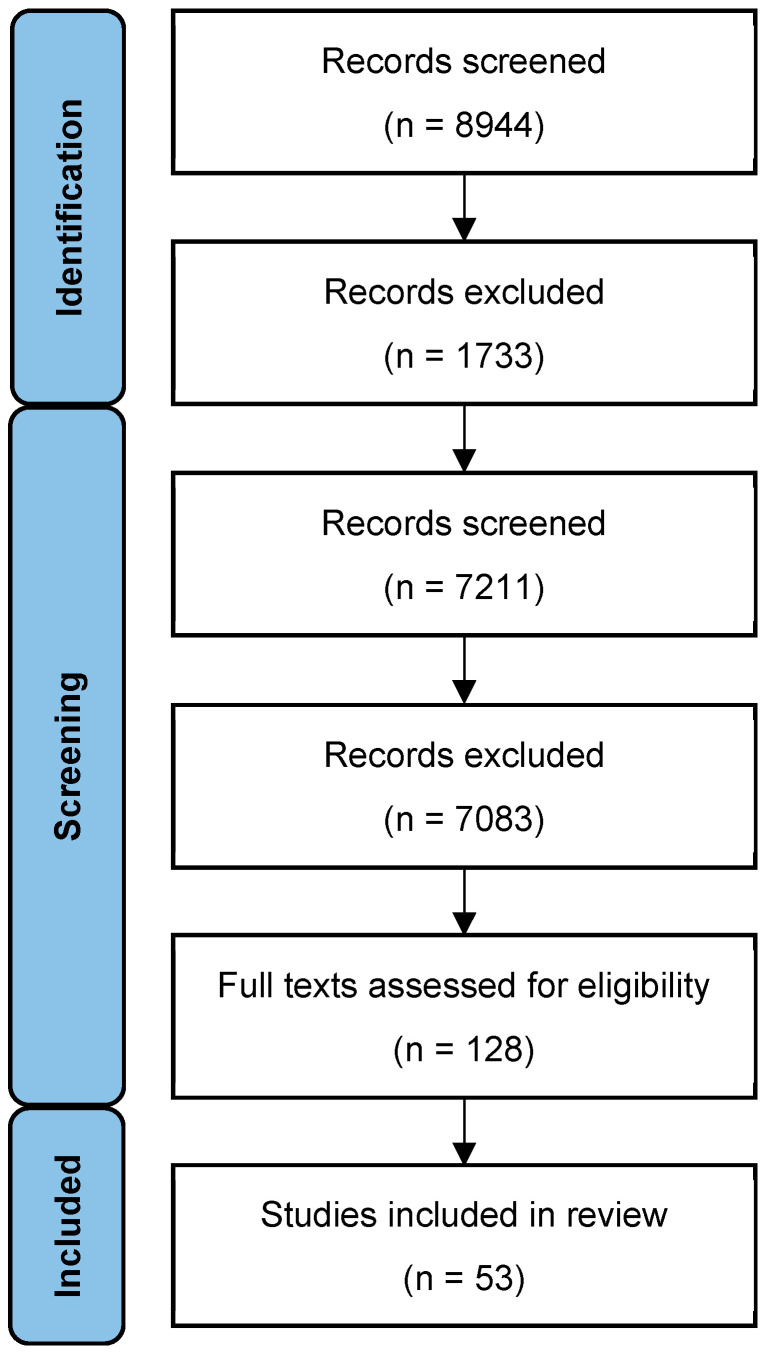
Flowchart of the study selection.

A search of the relevant electronic databases was conducted in April 2025 (PubMed, Ovid Medline, Embase, and the Cochrane Library) to retrieve all relevant articles using the keywords ‘Avascular necrosis “OR” AVN “OR” Osteonecrosis’ and ‘hip “OR” proximal femur’. The search was conducted by two authors (PR and VG) in an independent, unbiased manner. In case of disagreement, inclusion of a study was decided by consensus. In addition, the bibliographies of all identified relevant articles, including reviews, were searched for potentially relevant articles. The flowchart of study selection is presented in Figure 1.

### 2.3. Criteria of Eligibility

Inclusion criteria for the selection of studies included the following: (1) studies reporting on the outcomes of adult patients (age > 18) undergoing a proximal femoral osteotomy for the treatment of avascular necrosis; (2) more than 10 patients were included; and (3) the full text article was available in the English language. Exclusion criteria involved were the following: (1) studies which reported on multiple treatments whereby the data for the osteotomy cohort could not be isolated; and (2) studies where inadequate data were made available.

### 2.4. Extraction of Data

Each citation was reviewed for eligibility. Citations were initially reviewed on the basis of title and abstract. The remaining manuscripts were obtained and reviewed. Relevant information including authorship, publication year, study type, aetiology, operative details, post-operative rehabilitation regimen, peri-operative complications, radiological progression of disease, revision procedures including conversion to total hip replacement, and patient reported outcomes were recorded. Qualitative results were summarised and presented in tables. Statistical comparisons were not made between studies due to the heterogeneity in the data presented between studies.

### 2.5. Assessment of Risk of Bias

The quality of the included studies was assessed using the “NIH Quality Assessment Tool for Case Series Studies” (https://www.nhlbi.nih.gov/health-topics/study-quality-assessment-tools, accessed 27 May 2025) [15]. Using this tool, the rater assigns a three-level quality score of “good”, “fair”, or “poor”; based on the consideration of 9 items. The good quality indicates “low” risk of bias, the fair quality “moderate” risk of bias, and the poor quality indicates “significant” risk of bias (Table 1).

### 2.6. Data Synthesis and Analysis

Once the data extraction was complete, the data was transcribed into Microsoft Excel (Microsoft Excel, Version 16.78, Microsoft, Redmond, WA, USA) and analysed. Data were presented as absolute values and percentages. Weighted means were used to synthesise the data, with weights assigned based on the study sample size. Due to the high number of low-to-moderate quality studies with significant data heterogenicity, it was not possible to perform a meta-analysis. Consequently, a qualitative systematic narrative review was conducted to synthesise and present the available evidence.

## 3. Results

The initial search strategy identified 8944 citations once duplicates were removed. Following exclusions based on the information available in the title and abstract, 128 full texts were retrieved for review. Fifty-three studies were deemed to meet the inclusion criteria (forty-nine retrospective series, four prospective series), and were included in the review [16,17,18,19,20,21,22,23,24,25,26,27,28,29,30,31,32,33,34,35,36,37,38,39,40,41,42,43,44,45,46,47,48,49,50,51,52,53,54,55,56,57,58,59,60,61,62,63,64,65,66,67,68].

Across the 53 included studies, a total of 2686 osteotomies were performed in 2343 patients. The study population was 70% male with an average age of 37 (range 15–77) at the time of operation. The most common reason for AVN was the use of corticosteroids (647 patients) followed by alcohol consumption (560 patients) and idiopathic disease (407 patients). Aetiology is summarised in Table 2. Where classification was provided, the majority of studies utilised the Ficat classification system, with most cases in stage 3 (475), followed by stage 2 (287) (Table 3). Table 4 summarises the details of osteotomy type, with the most commonly performed type consisting of a rotational osteotomy (1534 patients), followed by varus osteotomy (440 patients). In most cases the osteotomy was fixed with screws (952 patients), followed by a sliding hip screw (765 patients) or a blade plate (672 patients).

Thirty-three studies commented on the post-operative protocol employed with six studies [28,47,50,55,63,66] placing patients in traction (average duration traction 2.7 weeks; range 1–4 weeks), and 13 mandating bed rest (average 3.6 weeks; range 0.3–8 weeks) [28,30,33,36,41,47,49,50,54,55,63,64,66]. A total of 5 studies asked patients to be non-weight bearing (NWB) prior to commencing full weight bearing (FWB) (average duration 14.2 weeks, range 6–23 weeks) [29,44,47,51,58], 10 studies asked patients to be partial weight bearing (PWB) prior to FWB (average duration 13.4 weeks, range 4–26 weeks) [19,21,22,23,26,41,42,54,61,68], whilst 18 instructed staged mobilisation with a period of NWB followed by PWB prior to commencing FWB (average duration NWB 8.4 weeks, range 3–26 weeks; average duration PWB 18.2 weeks, range 6–44 weeks) [20,24,27,28,30,32,33,34,49,50,51,52,53,55,64,65,67]. Overall patients spent 22 weeks (range 6–56 weeks) with restricted weight bearing prior to commencing FWB.

Follow up averaged 87 months (range 5–218 months). Complications were reported in 220/1520 (14.5%) hips where this data was provided [16,17,18,19,20,21,22,24,25,26,28,29,32,33,35,36,37,39,40,41,42,43,44,45,48,49,50,52,53,54,55,56,57,59,60,61,62,63,64,65,67,68]. The most common complication was delayed union (42 patients), fracture (40 patients), and fixation failure (33 patients). Complications are summarised in Table 5.

**Figure 2:**
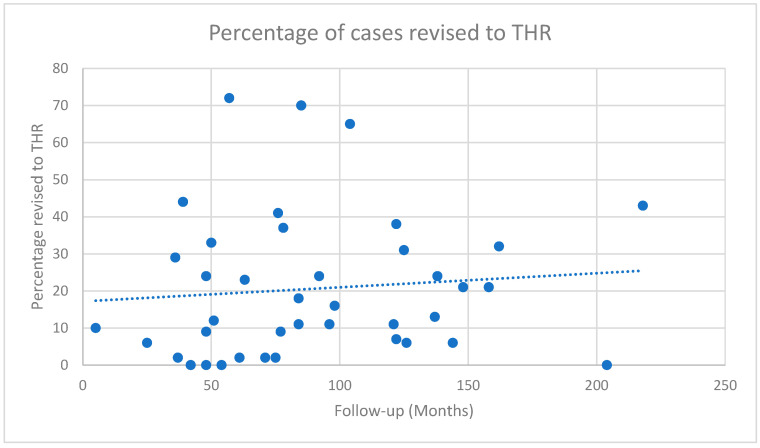
Percentage of Osteotomy cases revised to THR. Each dot represents the percentage of cases revised in each study at their final follow-up duration. The dotted line represents the line of best fit of all the studies.

Radiological progression of AVN was commented upon in 38 studies, with progression reported in 673/1684 hips (40%) [16,17,18,19,20,22,23,24,26,28,29,30,32,33,34,35,36,37,38,39,40,41,42,45,46,48,49,50,51,52,53,54,55,58,59,60,61,62,63,65,66,67,68]. Revision procedures were required in 23% of cases, with revision to THR required in 20.3% (mean time to revision 75.4 months, range 20–132 months; Figure 2). There was no significant temporal association with the requirement for revision to THR, although there was a gradual trend towards a higher revision rate with longer durations of follow-up.

**Figure 3:**
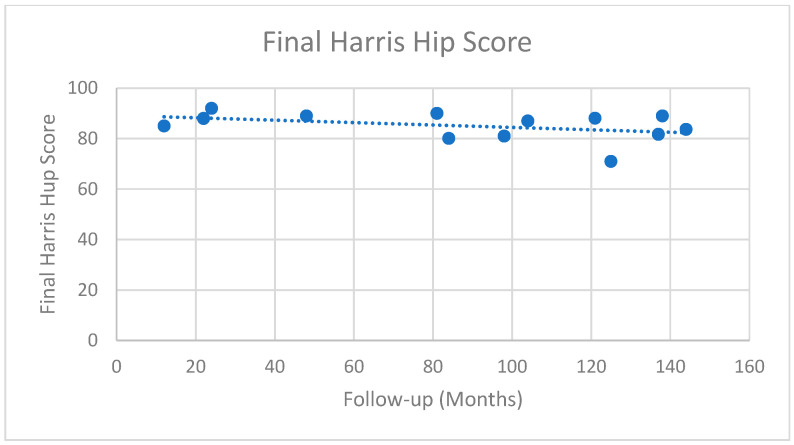
Final Harris Hip Score. Each dot represents the Harris Hip Score in each study at their final follow-up duration. The dotted line represents the line of best fit of all the studies.

**Figure 4:**
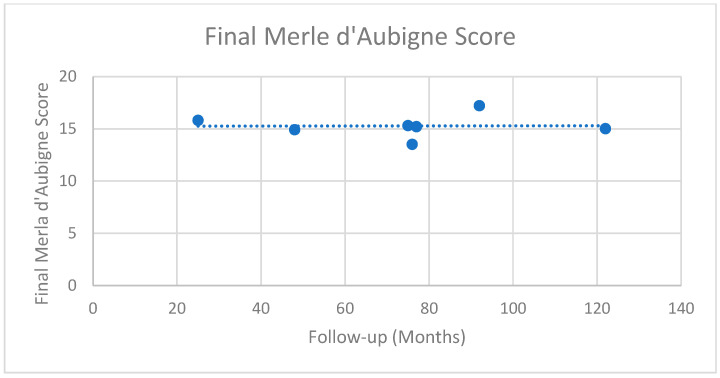
Final Merle d’Aubigne score. Each dot represents the Merle d’Aubigne Score in each study at their final follow-up duration. The dotted line represents the line of best fit of all the studies.

PROMS were recorded in 31 studies, including 1416 patients (Table 6) [16,17,18,19,20,21,22,23,24,25,26,27,28,29,30,31,33,34,36,37,38,39,40,41,43,44,45,46,47,48,50,51,52,53,55,56,58,61,63,64]. The Harris Hip Score (HHS) was the most commonly employed PROM, utilised in 16 series (745 patients) and measured at an average of 102 months (Figure 3, range 12–216 months) [16,18,20,22,23,25,28,29,30,34,36,39,40,52,58,61]. Across these studies, the average weighted improvement was 26 points, increasing from 58.3 pre-operatively to 84.4 at final follow-up. Duration of follow-up did not result in a significant change in the final HHS, with an average HHS of 87 in patients followed-up for between 12–60 months, 81.5 in patients followed-up between 60–120 months, and 84.2 in patients followed-up for >120 months. Merle d’Aubigne was the second most commonly utilised PROM, with numeric data available in seven series (418 patients) measured at an average of 74 months (Figure 4, range 25–122 months) [19,24,26,37,44,55,63]. In these patients there was a mean increase from 12.8 to 15.6, representing a transition from a poor to a good outcome.

## 4. Discussion

Osteotomy in the management of AVNFH provides a two-fold benefit, both relocating the necrotic segment away from the weight bearing area and improving the vascularity to the necrotic area. Osteotomy is indicated when there is evidence of subchondral fracture or early collapse, with core decompression more appropriate in the pre-collapse stage, and arthroplasty indicated when secondary degenerative changes have occurred. None the less, the usage of osteotomy is reducing, with many preferring to instead turn to arthroplasty in diseases with early collapse and without secondary arthritis. This is potentially disadvantageous, in the young adult population, as it exposes the patient to a potential 10-year risk of re-operation of 16.3% when undergoing THR for any cause. This is particularly important given that the revision rate of patients undergoing THR for AVN is already known to be higher than that for OA, although promising early results are seen with uncemented ceramic on polyethylene systems [69,70,71].

This study presented data on 2686 hips in 2343 patients across 53 studies, with a median volume of 38 hips per study (IQR 20–54). Predisposing factors towards AVN were as per the published literature, predominantly corticosteroid and alcohol use [72]. Where a classification was provided, the majority of cases were collapsed (Ficat grade 3), where alternative head-preserving surgical techniques such as core decompression are associated with a failure rate of nearly double that of osteotomy [73]. Greater than two thirds of our study population underwent a rotational osteotomy, whilst a further 18% underwent a varus-producing osteotomy.

Progression to radiological arthrosis following osteotomy was seen in 40% of cases; however, just over half of these patients’ (20.3%) required conversion to a THR at the median follow-up of 87 months. Unfortunately, few studies broke down the rates of THR by AVN stage, and, therefore, comparisons between pre-collapse and post-collapse disease could not be made. Within the osteotomy population post-operative complications were seen in 14.5%, most commonly delayed union, fracture, and fixation failure. Few studies reported on the correlation between necrotic angle and success of osteotomy. Classically, a necrotic angle of >200° has been associated with radiological progression [10]. Only one study within this review commented on necrotic angle as a predictor of treatment success, noting an average necrotic angle of 190° in their treatment successes compared to an average angle of 240° degrees in their treatment failures [60]. In their patient population, Fuchs et al. reported an average necrotic angle of 248°, and yet reported a rate of conversion to THR of just 13% at 10 years, increasing to 32% at 15 years [41]. Lee et al. reported on 91 hips with necrotic angles ranging from 195° to 260°, reporting secondary collapse in 36% and conversion to THR in 23% at a mean of 7.5 years [19].

Whilst necrotic angle is associated with radiological progression of arthrosis, it has not been demonstrated to predict requirement for conversion to THR, and, therefore, it should be used with caution when deciding if a patient should undergo arthroplasty or osteotomy [74]. The presence of radiological arthrosis in the absence of symptoms has been well documented in the hip osteoarthritis (OA) population, with up to 80% of patients with radiological changes not demonstrating any symptoms [75]. Therefore, the collection of PROMS is crucial to fully understanding the functional outcome obtained by these patients.

In reporting PROMS, the most commonly employed outcome measure was the Harris Hip Score (HHS), a scoring system that provides a score out of 100 across the four domains of pain, function, activity and range of movement [76]. Within the healthy population the average HHS is 95.6, higher than the 87–92 seen following THR for OA [77,78,79,80]. Outcomes of THR for AVN report similar HHS, ranging from 85 to 94 [71]. Within the study population the average Harris Hip Score measured at 8.5 years was 84.4, representing an improvement of 26 points compared to pre-op.

Whilst the HHS is lower than has been seen within the overall arthroplasty population, this may be explained by the higher overall functional demand and expectations of younger patients, with the prior literature demonstrating lower HHS in this patient cohort. In their randomised controlled trial of THR and hip resurfacing in younger adult patients with hip osteoarthritis, Costa et al. reported that one year post THR the average HHS in their cohort was just 82.3 [81]. Similar results were described by Lombardi et al., describing a mean HHS of 82.4 at two years in their series of 643 patients aged 55 and younger [82]. The HHS has a minimally clinically important difference of seven; therefore, the outcomes seen within this review would suggest that the functional outcome achieved with osteotomy is similar to that achieved with THR within the literature, although a comparative study would be required to formally demonstrate this [83].

There are few comparative studies directly comparing THR with osteotomy. Osawa et al. compared outcomes in patients under the age of 50, establishing that there was no difference in PROMs at 11.5 years between the two techniques, although the pre-operative PROMS were significantly higher in the osteotomy group (pre-op HHS 70.2 in osteotomy group vs. 59.6 in the THR group) [84]. Kubo et al. similarly compared PROMS in 20 patients undergoing anterior rotational osteotomy (9 patients) or THR (11 patients) for stage 3 AVN demonstrating equivalent Oxford hip scores at a mean of 2.5 years (38.4 in osteotomy group vs. 40.3 in THR group) [21]. Again, however, pre-operative PROMS were significantly lower in the THR group. Similar results have also been published by Seki et al. and Kang et al., both of whom noted equivalent outcomes in the SF-36 when comparing THR and osteotomy [31,85].

One argument against the use of osteotomy is the potential future difficulty of performing a THR due to the abnormal femoral anatomy. THR following osteotomy is associated with longer operative times, higher blood loss, and a higher incidence of intra-operative periprosthetic fracture [86]. Elevated risks persist in the post-operative period with a recent systematic review by Goh et al. demonstrating a relative risk of 3.37 for surgical site infection and a poorer 5-year implant survivorship, although there was no significant difference in the incidence of post-operative periprosthetic fracture, dislocation, aseptic loosening, or the need for revision surgery [86]. Within this study, THR following osteotomy was also associated with a 5.58-point reduction in the Harris Hip Score compared to those undergoing primary THR without prior surgery.

The previous literature has highlighted an increased rate of intra-operative fracture, and also highlighted key complications including nerve palsies following excessive lengthening during the arthroplasty procedure [87,88]. Nonetheless it did not report a difference in implant survivorship, in contrast to Goh et al., suggesting this requires further examination. Whilst outcomes of arthroplasty may be altered following osteotomy, 20.3% of patients required THR following osteotomy in the presented study, and, therefore, one should be cautious in avoiding offering this hip-preserving procedure on the basis of the potential for a future complex THR, although the patient should be made aware of the complication profile of future THR prior to embarking on osteotomy.

There are several limitations that should be taken into consideration when reviewing the presented systematic review. This review documents a series spanning almost 50 years, with evolution in techniques and significant heterogeneity in the presentation of both classification data and the surgical technique performed. The majority are retrospective in nature and are, therefore, susceptible to the inherent biases and confounders encountered with this research type. Using the NIH quality assessment tool for case series studies, 16 studies were rated “good”, 20 were rated “fair”, and 17 were rated “poor”. Furthermore, several series were undertaken in Asian populations and, therefore, given anthropometric differences between Asian and other populations, the results may not be comparable in non-Asian populations.

## 5. Conclusions

Osteotomy remains a valid femoral head-preserving technique in the armamentarium against avascular necrosis of the femoral head. Patients can be informed that conversion to THR is required in 20.3% of cases at a mean of seven years, and that there is a complication rate of 14.5%. PROMS are comparable to those achieved with THR; however, further comparative trials would be needed to be able to accurately compare the two techniques. Further research areas in patient selection for osteotomy should focus on success rates dependent on the size and location of the necrotic area and also assess the outcomes and survivorship of patients undergoing THR with modern implants and techniques, where outcomes would be expected to be improved.

## Figures and Tables

**Table 1:** Risk of Bias Assessment.

Author	Year	Quality of Study	Risk of Bias
Kubo [16]	2019	Good	Low
Kawano [17]	2018	Good	Low
Kubo [18]	2017	Good	Low
Lee [19]	2017	Good	Low
Morita [20]	2017	Good	Low
Kubo [21]	2016	Good	Low
Okura [22]	2016	Good	Low
Sonoda [23]	2015	Fair	Moderate
Hamanishi [24]	2014	Fair	Moderate
Ito [25]	2012	Good	Low
Ha [26]	2011	Good	Low
Motomura [27]	2010	Good	Low
Biswal [28]	2009	Good	Low
Yoon [29]	2008	Good	Low
Sugioka [30]	2008	Good	Low
Seki [31]	2008	Fair	Moderate
Ikemura [32]	2007	Good	Low
Atsumi [33]	2006	Poor	High
Nakamura [34]	2005	Poor	High
Onodera [35]	2005	Fair	Moderate
Rijnen [36]	2005	Good	Low
Hisatome [37]	2004	Fair	Moderate
Langlais [38]	2004	Fair	Moderate
Sakano [39]	2004	Fair	Moderate
Drescher [40]	2003	Fair	Moderate
Fuchs [41]	2003	Fair	Moderate
Pavlovcic [42]	2002	Poor	High
Schneider [43]	2002	Fair	Moderate
Gallinaro [44]	2001	Poor	High
Koo [45]	2001	Good	Low
Lengsfeld [46]	2001	Fair	Moderate
Nakai [47]	2000	Poor	High
Inao [48]	1999	Fair	Moderate
Atsumi [49]	1997	Poor	High
Iwasada [50]	1997	Fair	Moderate
Langlais [51]	1997	Poor	High
Mont [52]	1996	Fair	Moderate
Grigoris [53]	1995	Fair	Moderate
Dean [54]	1993	Poor	High
Sugano [55]	1992	Fair	Moderate
Hotokebuchi [56]	1992	Poor	High
Melzer [57]	1992	Poor	High
Kinnard [58]	1990	Poor	High
Gottschalk [59]	1989	Poor	High
Jacobs [60]	1989	Poor	High
Maistrelli [61]	1988	Fair	Moderate
Masuda [62]	1988	Poor	High
Saito [63]	1988	Fair	Moderate
Eyb [64]	1987	Poor	High
Tooke [65]	1987	Fair	Moderate
Sugioka [66]	1984	Fair	Moderate
Imizcoz [67]	1984	Poor	High
Kotz [68]	1981	Poor	High

**Table 2:** Aetiology of Avascular Necrosis of the Femoral Head.

Aetiological Factor	N (%)
Steroid	647 (34%)
Alcohol	560 (29%)
Idiopathic	407 (21%)
Trauma	135 (7%)
Smoking	54 (3%)
Hyperlipidemia	49 (3%)
Pregnancy	14 (<1%)
Congenital dislocation	9 (<1%)
Perthes	8 (<1%)
Heavy metal	8 (<1%)
Fat embolism	4 (<1%)
Caisson	2 (<1%)
Systemic Lupus	2 (<1%)
Diabetes	2 (<1%)
Gout	1 (<1%)
Radiation	1 (<1%)
Gauchers	1 (<1%)
Chemotherapy	1 (<1%)

**Table 3:** Classification of Avascular Necrosis of the Femoral Head.

Classification (Number of Studies)	N (%)
Ficat (20)	
Stage 1 Stage 2 Stage 3 Stage 4	30 (4%) 287 (35%) 475 (58%) 24 (3%)
Japanese Investigation Committee of Health and Welfare (9)	
Stage 1 Stage 2 Stage 3 Stage 4	1 (<1%) 68 (20%) 260 (77%) 7 (2%)
Type A Type B Type C Type D	41 (10%) 27 (7%) 339 (83%) 0 (0%)
ARCO (5)	
Stage 1 Stage 2 Stage 3 Stage 4	0 (0%) 25 (11%) 182 (80%) 21 (9%)
Steinburg (2)	
Stage 1 Stage 2 Stage 3 Stage 4	0 (0%) 42 (51%) 35 (42%) 6 (7%)
JOA Hip Score (2)	
Stage 1 Stage 2 Stage 3 Stage 4	1 (<1%) 115 (34%) 148 (44%) 72 (21%)
Merle d’Aubigiune (1)	
Stage 1 Stage 2 Stage 3	2 (13%) 13 (81%) 1 (6%)
Not Recorded (14)	

**Table 4:** Osteotomy type and Fixation strategy.

Osteotomy Details	N (%)
Osteotomy type	
Rotation Flexion Varus Valgus Flexion + Varus Flexion + Valgus Rotation + Varus Rotation + Valgus	1534 (64%) 41 (2%) 440 (18%) 150 (6%) 120 (5%) 32 (1%) 40 (2%) 32 (1%)
Fixation type	
Screws Sliding hip screw Blade plate	952 (40%) 765 (32%) 672 (28%)

**Table 5:** Complications following Osteotomy for Avascular Necrosis of the Femoral Head.

Complication	N (%)
Delayed union	42 (19%)
Fracture	40 (18%)
Fixation failure	33 (15%)
Non-union	32 (15%)
Deformity	28 (13%)
Deep infection	21 (10%)
DVT/PE	8 (4%)
Haematoma	6 (3%)
Nerve injury	4 (2%)
Surgical site infection	4 (2%)
Poor wound healing	1 (<1%)
Heterotopic ossification	1 (<1%)

**Table 6:** Patient-Reported Outcomes following Osteotomy for Avascular Necrosis of the Femoral Head.

PROM (Number of Studies; Hips)	Outcome
Harris Hip Score (16; 745) [16,18,20,22,23,25,28,29,30,34,36,39,40,52,58,61]	
Pre-op Post-op Average change	58.3 84.4 26.1
Merle d’Aubigne Numeric Score (7; 418) [19,24,26,37,44,55,63]	
Pre-op Post-op Average change	12.8 15.1 2.3
Merle d’Aubigne Outcome Rating (6; 137) [33,37,38,41,46,51]	
Excellent Good Fair Poor	61 (45%) 35 (26%) 18 (13%) 23 (17%)
Oxford Hip Score (2; 115) [17,21]	
Post-op score	37.3
UCLA Activity Scale (1; 95) [17]	
Post-op score	5
Pain Catastrophizing Score (1; 95) [17]	
Post-op score	43

## Data Availability

Data available upon request.

## Abbreviations

The following abbreviations are used in this manuscript:

AVNFH	Avascular Necrosis of the Femoral Head
FWB	Full Weight Bearing
HHS	Harris Hip Score
IQR	Inter Quartile Range
NIH	National Institute for Health
MRI	Magnetic Resonance Imaging
NWB	Non-Weightbearing
OA	Osteoarthritis
PRISMA	Preferred Reporting Items for Systematic Reviews and Meta-Analysis
PROMS	Patient Reported Outcome Measures
PWB	Partial Weightbearing
THR	Total Hip Replacement
